# Potential Functional Variants in DNA Repair Genes Are Associated with Efficacy and Toxicity of Radiotherapy in Patients with Non-Small-Cell Lung Cancer

**DOI:** 10.1155/2020/3132786

**Published:** 2020-06-24

**Authors:** Zhiguang Yang, Zhaoyu Liu

**Affiliations:** Department of Radiology, Shengjing Hospital of China Medical University, Shenyang 110000, China

## Abstract

**Background:**

Lung cancer is one of the leading causes of cancer-related deaths. Radiotherapy, either alone or with chemotherapy, is still the primary treatment for patients with non-small-cell lung cancer (NSCLC). There are variations in how patients with NSCLC respond to radiotherapy and how toxic the therapy is. DNA repair gene polymorphisms are related to cancer development; however, their association with radiotherapy outcomes remains unknown. We hypothesized that gDNA repair gene variation could affect the efficacy and toxicity of radiotherapy in patients with NSCLC.

**Methods:**

A total of 486 histologically confirmed patients with NSCLC were recruited from the Shengjing Hospital of China Medical University from July 2015 to September 2019. Eleven potentially functional single nucleotide polymorphisms (SNPs) in four DNA repair genes (*XRCC1*, *XRCC2*, *XPD*, and *MSH2)* were genotyped in these patients. A multiple factor logistic regression analysis was used to assess the association between these SNPs and the efficacy and toxicity of radiotherapy.

**Results:**

Three SNPs, rs25487 (*XRCC1*), rs3218556 (*XRCC2*), and rs13181 (*XPD*), were all significantly associated with the efficacy of radiotherapy. The allele frequencies of the rs25487 CC genotype (OR = 0.457, 95% CI = 0.259–0.804, *p*=0.006) and the rs3218556 AG or AA genotypes (AG genotype: OR = 0.664, 95% CI = 0.442–0.999, *p*=0.049; AA genotype: OR = 0.380, 95% CI = 0.181–0.795, *p*=0.008) were both significantly higher in the response group than in the nonresponse group. For rs13181, the radiotherapy efficacy was associated with the heterozygous genotype GT (OR = 1.663, 95% CI = 1.057–2.614,*p*=0.027). Statistically significant associations between radiation-induced toxic reactions and rs25487 (*XRCC1*), rs3218556 (*XRCC2*), and rs13181 (*XPD*) were also observed. The rs13181GT genotype was associated with lower toxic reactions than the TT genotype (OR = 1.680, 95% CI = 1.035–2.728,*p*=0.035).

**Conclusions:**

The variants rs25487 (*XRCC1*), rs3218556 (*XRCC2*), and rs13181 (*XPD*) all contribute to the efficacy and toxicity of radiotherapy in patients with NSCLC. Our findings may clarify the predictive value of DNA repair genes for prognosis in patients with NSCLC after radiotherapy. Further investigation of more genes and samples should be performed to confirm our findings.

## 1. Introduction

Lung cancer accounts for a large proportion of cancer patients worldwide and is the leading cause of cancer-related deaths [1]. There were 9.6 million cancer-related deaths in 2018, 18.4% of which were caused by lung cancer [2]. In China, about 28% and 23% of cancer-related death in 2012 occurred as a result of lung cancer in men and women, respectively. Non-small-cell lung cancer (NSCLC), including lung adenocarcinoma (LUAD) and lung squamous cell carcinoma (LUSC), accounts for approximately 85% of all lung cancers [3]. With a better understanding of tumor biology, there has been a breakthrough in the treatment of lung cancer. However, radiotherapy, either alone or with chemoradiotherapy, is still the primary treatment or palliative care for many patients, especially for locally advanced NSCLC patients who cannot be surgically resected. There is often a significant difference in treatment effects and toxic reactions in NSCLC patients undergoing standard radiotherapy [4, 5]. Genetic variations between individuals or tumors are the main reasons for the differences in sensitivity to radiotherapy [6].

Ionizing radiation leads to cell death mainly by creating double-strand breaks (DSB) [7] or through damage to cell membranes [8]. Studies have shown that DNA repair genes, such as *ERCC1* [9], *XRCC1* [10], and *XPA* [11], play a key role in the different repair processes that are invoked in cells following DNA damage. There are several DNA repair pathways in the human body, of which the nucleotide excision repair (NER), mismatch repair (MMR), and homologous recombination (HR) systems repair the damaged DNA after the formation of cross-link chains, whereas the base excision repair (BER) pathway repairs it before the formation of cross-link chains. *XRCC1* and *XRCC2* are involved in the BER and HR pathways, respectively, and have been associated with the occurrence of cancer [12, 13]. *MutS homolog 2 (MSH2)*, a key component of the MMR pathway, plays an important role in the development of neoplastic diseases [14]. The *Xeroderma pigmentosum group D* (*XPD*) gene, another important DNA repair gene, has been reported to contribute to the risk of human cancer [15].

Although the relationship between polymorphisms in DNA repair genes and the development of cancer has been well explored in previous studies, few reports have investigated the interrelationship between gene polymorphisms and radiosensitivity or radiotherapy toxicity in patients with NSCLC. To explore the possible association between DNA repair gene variations and radiotherapy sensitivity and associated toxicity, we evaluated eleven single nucleotide polymorphisms (SNPs) in four DNA repair genes (*XRCC1*, *XRCC2*, *XPD*, and *MSH2*).

## 2. Materials and Methods

### 2.1. Study Population

In this study, a total of 486 histologically confirmed NSCLC patients were recruited from the Shengjing Hospital of China Medical University from July 2015 to September 2019. The detailed characteristics of these patients are described in Table 1. The inclusion criteria were as follows: (1) patients who were diagnosed as having primary NSCLC and not eligible for surgery; (2) patients who underwent a lung biopsy and had a confirmed histopathological diagnosis of NSCLC; (3) patients who received a standard dose of radiotherapy; (4) patients who had no recurrent disease; (5) patients who had no other malignant tumors or history of radiotherapy. All participants or family members signed an informed consent form before blood collection and analysis. This study was approved by the ethics committee of the Shengjing Hospital of China Medical University.

### 2.2. Radiotherapy Treatment and Evaluation

All patients were treated with three-dimensional conformal radiation therapy (3D-CRT) or intensity modulated radiation therapy (IMRT), with a total radiation dose of 50–70 Gy. Followups were conducted on all patients three months after radiotherapy, and their response to treatment was assessed using computed omography (CT) according to the Response Evaluation Criteria in Solid Tumors (RECIST) guidelines. There were four categories of response defined: complete response (CR), partial response (PR), stable disease (SD), and progressive disease (PD) [16]. In the present study, CR and PR were grouped as “responders,” whereas SD and PD were grouped as “nonresponders.” Radiation-induced toxic reactions were graded according to the Radiation Therapy Oncology Group or European Organization for Research and Efficacy of Cancer (RTOG/EORTC) guidelines. Patients with grade 0 and grade 1 reactions were considered to have “low-toxic reactions” and those with grades 2–5 reactions, “high-toxic reactions.”

### 2.3. Selection of SNPs and Genotyping

Four DNA repair genes, *XRCC1*, *XRCC2*, *MSH2,* and *XPD*, which had previously been shown to be positively associated with the development of cancer, were selected for analysis. SNP genotypes were downloaded from the 1000 Genomes project (https://www.internationalgenome.org/) and analyzed using Haploview 4.2 software (https://www.broadinstitute.org/haploview/haploview). Candidate SNPs which met the following criteria were included: (1) SNPs that had a minor allele frequency (MAF) > 0.1 in Han Chinese in Beijing (CHB); (2) SNPs with a potential function, such as missense variations causing amino acid changes or were present in the 5′ or 3′ untranslated regions (UTRs) that could affect transcription factor binding site (TFBS) activity; (3) SNPs that have been reported in previous association studies. As a result of using these selection criteria, a total of 11 potentially functional SNPs in DNA repair genes were selected. These included 4 *XRCC1* SNPs (rs25487 (exon 10), rs25489 (exon 9), rs1799782 (exon (6) and rs3213245 (5′ UTR)), 3 *XRCC2* SNPs (rs3218556 (3′ UTR), rs3218544 (3′ UTR), rs3218385 (5′ UTR)), 2 *MSH2* SNPs (rs2303424 (exon 16) and rs2303425 (5′ UTR)), and 2 *XPD* SNPs (rs13181 (exon 10) and rs238419 (3′ UTR)).

Genomic DNA from all patients was extracted using a TIANamp Genomic DNA Kit (Tiangen Biotech, Beijing, China). Genotyping was performed using the TaqMan methodology and an Applied Biosystems 7500 FAST Real-Time PCR System (Applied Biosystems, Foster City, CA, USA) according to the manufacturer's instructions. The predesigned SNP-genotyping assay mixture, containing the PCR primers and probes, were supplied by Applied Biosystems. The PCR amplification mix was prepared as follows: 25 *μ*L of master mix (Applied Biosystems), 10 *μ*L of DNA, and 15 *μ*L of ddH_2_O. Amplification was performed under the following conditions: 50°C for 2 min, 95°C for 10 min, 40 cycles of 95°C for 15 sec, 60°C for 1 min. Three negative controls (no DNA) and three positive controls in each 96-well plate were used to ensure the accuracy of the PCR amplification. In addition, 10% of randomly repeated samples were used for quality control.

### 2.4. Statistical Analysis

Statistical analyses were performed using SPSS 22.0 software (SPSS, Chicago, IL, USA). The Hardy-Weinberg equilibrium was used to assess that all SNPs met the group representation. A multiple factor logistic regression was applied to assess the association between SNPs and efficacy of radiotherapy as well as radiation-induced toxicity reaction after adjusting for age, gender, smoking history, cancer histology, family history, and chemotherapy. A *p* < 0.05 was considered statistically significant.

## 3. Results

### 3.1. Characteristics of Patients and Clinical Outcomes

The clinical characteristics and demographics of the 486 NSCLC patients are described in Table 1. The proportion of male patients (67.5%) was greater than double the proportion of female patients (32.5%). The median age of all patients was 62 (ranging from 33 to 84). About half of the patients had a history of smoking. There were 307 (63.2%) cases of squamous cell carcinoma and 179 (36.8%) cases of adenocarcinoma. Seventy-six patients (15.7%) had a family history of cancer and 383 (78.8%) had been treated with chemotherapy. The response rate to radiotherapy was 48.1%.

### 3.2. Associations between Candidate SNPs and Efficacy of Radiotherapy

The associations between candidate SNPs and the response to radiotherapy in NSCLS patients are shown in Table 2. All the candidate SNPs reached equilibrium according to the Hardy-Weinberg equilibrium test (*p* > 0.05, data not shown). The SNPs rs25487 (*XRCC1*), rs3218556 (*XRCC2*), and rs13181 (*XPD*) all showed a significant association with the efficacy of radiotherapy. The allele frequency of rs25487 CC genotype (OR = 0.457, 95% CI = 0.259–0.804,*p*=0.006) and the rs3218556 AG or AA genotypes (AG genotype: OR = 0.664, 95% CI = 0.442–0.999,*p*=0.049; AA genotype: OR = 0.380, 95% CI = 0.181–0.795, *p*=0.008) were significantly higher in the response group than in the nonresponse group. For rs25487, although the response rate between the CT and CC genotypes was not statistically significant (*p*=0.078), a higher response rate was also observed when comparing the CT + CC genotype with the CC genotype, using a dominant model. For rs13181, a better radiotherapy efficacy was associated with the heterozygotic genotype GT (OR = 1.663, 95% CI = 1.057–2.614, *p*=0.027). There were no significant associations between the other SNPs and the efficacy of radiotherapy.

### 3.3. Associations between Candidate SNPs and Radiation-Induced Toxic Reactions

The associations between candidate SNPs and radiation-induced toxic reactions are shown in Table 3. A statistically significant association between radiation-induced toxic reactions and rs25487 (*XRCC1*), rs3218556 (*XRCC2*), and rs13181 (*XPD*) was observed. For rs25487, the genotypes CT, TT, and CT + TT were associated with a severe toxic reaction compared to the CC genotype (all, *p* < 0.05). For rs3218556, the ORs for cases with the AG, AA, and AG + AA genotypes compared with homozygous CC genotype were 0.605 (95% CI = 0.396–0.924, *p*=0.019), 0.279 (95% CI = 0.116–0.675, *p*=0.003), and 0.540 (95% CI = 0.362–0.805, *p*=0.002), respectively. The rs13181GT genotype was associated with lower toxic reactions compared with the TT genotype (OR = 1.680, 95% CI = 1.035–2.728, *p*=0.035).

## 4. Discussion

NSCLC is the leading cause of cancer-related death. Radiotherapy is an important treatment for NSCLC patients, especially for advanced NSCLC patients. However, there are significant differences in the efficacy of radiotherapy as well as in the incidence rate for radiation-induced toxic reactions. Identification of the key determinants that affect efficacy and toxicity is of paramount importance for the efficacy of radiotherapy in patients with NSCLC. Although numerous studies have reported that genetic polymorphisms in DNA repair genes are related to the development of cancer, their association with the outcomes of radiotherapy remains unknown. In the present study, we demonstrated that rs25487 (*XRCC1*), rs3218556 (*XRCC2*), and rs13181 (*XPD*) were associated with the efficacy and toxicity of radiotherapy in patients with NSCLC.

The potential functional variations of the four DNA repair genes were listed in Supplementary Table 1. The variations of rs25487 (c.1196A > G, p. Gln399Arg), rs25489 (c.839G > A, p. Arg280His), rs1799782 (c.580C > T, p. Arg194Trp) of *XRCC1* gene, rs2303424 (c.2744A > G, p. Gln915Arg) of *MSH2* gene, and rs13181 (c.2251A > C, p. Lys751Gln) of *XPD* gene cause amino acid changes and then affect the biological function of the protein. Also, other variations located in the 5′ or 3′ UTRs may affect the transcription factor binding site (TFBS) activity and then influence the DNA repair gene expression. All the variants may impact the clinical outcome of radiotherapy through these two ways.

Many genetic variants that are involved in DNA damage repair and the regulation of oxidative stress are associated with radiotherapy outcomes [17]. *XRCC1*, an important component of BER, has been reported to be associated with an increased risk of NSCLC in nonsmoking female patients with a history of exposure to cooking oil mist [10]. Genetic polymorphisms in *XRCC1*-194 and *XRCC1*-399 are also related to the risk of NSCLC [18]. Wang et al. [19] have reported that the presence of *XRCC1* rs25489 had a significant impact on primary tumor efficacy at the end of radiotherapy and may act as a biomarker for the curative effect of radiotherapy. Zhai et al. [20] found that patients with nasopharyngeal carcinoma (NPC) carrying the *XRCC1* codon 399 Gln/Gln genotype had a higher rate of tumor regression after radiotherapy. Another study showed that, in 114 patients with NPC, the *XRCC1* rs25487 GA genotype was related with grade 3 dermatitis and grade 3 mucositis. In this study, a significant association was also observed between the rs25489 CC genotype and a higher response rate to radiotherapy as well as lower toxic reaction. Although three other SNPs (rs25489, rs1799782, and rs3213245) in *XRCC1* were also genotyped, no significant associations were observed. Due to the fact that *XRCC1* rs25487 is clearly important in radiation sensitivity and the resultant toxic reactions, it could be considered as a biomarker that can be used to predict the clinical outcomes of radiotherapy.

Three SNPs (rs3218556, rs3218544, and rs3218385) in *XRCC2*, which is involved in the HR pathway, were also evaluated for a possible association with the efficacy and toxicity of radiotherapy in patients with NSCLC. Of these, only rs3218385 showed a significant association with both the efficacy and toxicity of radiotherapy. Popanda et al. [7] suggested that there was no relationship between the risk of acute skin toxicity and *XRCC2* variations in patients with breast cancer receiving radiotherapy, which is inconsistent with our findings. Nevertheless, Yin et al. [21] genotyped six potentially functional SNPs in 228 patients with NSCLC who had been treated with definitive radiotherapy and found that *XRCC2* R188H SNPs was independent prognostic factor for overall survival. Qin et al. [22] also found that *XRCC2*-deficient cancer cells were more sensitive to irradiation *in vitro* and speculated that the inhibition of *XRCC2* expression or activity represents a potential therapeutic strategy for improving preoperative radiotherapy responses in patients with locally advanced rectal cancer. These results strongly suggest that *XRCC2* plays an important role not only in the development of the cancer but also in radiotherapy outcomes.

*MSH2* is an important MMR gene and several studies have suggested that variations in *MSH2* are associated with sensitivity to radiotherapy and disease progression in rectal cancer patients [23, 24]. However, the present study found that there was no association between two SNPs in this gene (rs2303424 and rs2303425) and the efficacy of radiotherapy. Xie et al. [25] also found that there was no correlation between survival time and *MSH2* gene expression levels in patients with NSCLC who were treated with chemotherapy, which is consistent with our results. SNPs in the *XPD* gene have been widely studied and suggested to be genomic markers to predict the response to radiation dose and potentially guide personalized radiotherapy [18, 26]. This study also discovered that *XPD* rs13181 was associated with the progression and outcomes of patients with NSCLC after radiotherapy. Patients carrying the rs13181 GT genotype who were treated with radiotherapy had a high level of toxic reaction coupled with low efficacy.

Despite the positive findings, there are also some limitations in this study. First, we were unable to explain how the DNA repair gene variations influenced the outcomes of radiotherapy. Secondly, too few SNPs were genotyped. We selected the potentially functional SNPs based on three criteria so that the SNPs did not cover all SNPs in the entire gene. Thirdly, our sample size is still too small. In this study, we failed to replicate the association between *MSH2* and NSCLC patients. This could be due to the genetic heterogeneity in different population and a small number of samples included. Fourthly, gene–gene and gene–environment interactions were not analyzed. Additionally, some other important genes, such as *ATM* gene of DNA-damage sensing [27], *ATG16L2* gene related to autophagy [28], and *RUNX3* [29] gene in the methionine metabolic pathway that have been reported to be associated with the outcomes of NSCLS, were not included in this study.

In conclusion, we have identified patients with NSCLC carrying the rs25487 (*XRCC1*), rs3218556 (*XRCC2*), and rs13181 (*XPD*) SNPs, which appear to contribute to the efficacy and toxicity of radiotherapy. Our findings may be helpful in understanding the predictive value of examining DNA repair genes in the prognosis of patients with NSCLC after radiotherapy. Further investigation of more genes and samples should be carried out to confirm our findings. In addition, more studies should be implemented to explain the underlying molecular mechanisms to explain how these polymorphisms affect the response to radiotherapy and prospective clinical trials in patients with NSCLC.

## Figures and Tables

**Table 1:** Clinical characteristics and demographics of the NSCLC patients.

Characteristic	NSCLC patients (*n* = 486)
Age (years)	62 (33–84)
Gender (%)	
Male	328 (67.5%)
Female	158 (32.5%)
Smoke history (%)	
Smokers	244 (50.2%)
Never smokers	242 (49.8%)
Histology (%)	
Squamous cell carcinoma	307 (63.2%)
Adenocarcinoma	179 (36.8%)
Family history of cancer (%)	
Yes	76 (15.7%)
No	410 (84.3%)
Chemotherapy (%)	
Yes	383 (78.8%)
No	103 (21.2%)
Response (%)	
Response (CR + PR)	234 (48.1%)
Nonresponse (SD + PD)	252 (51.9%)

**Table 2:** Association between candidate SNPs and efficacy of radiotherapy.

Gene		SNPs	Genotype	CR + PR	SD + PD	OR (95% CI)	*p*
*XRCC1*		rs25487	CC	130	111	Reference	Reference
			CT	81	98	0.706 (0.479–1.041)	0.078
			TT	23	43	0.457 (0.259–0.804)	**0.006**
			CT + TT	104	141	0.630 (0.440–0.901)	**0.011**
		rs25489	CC	190	203	Reference	Reference
			CT	42	47	0.955 (0.602–1.514)	0.844
			TT	2	2	1.068 (0.149–7.661)	0.947
			CT + TT	44	49	0.959 (0.610–1.509)	0.858
		rs1799782	GG	128	121	Reference	Reference
			AG	84	97	0.819 (0.558–1.201)	0.306
			AA	22	34	0.612 (0.339–1.105)	0.101
			AG + AA	106	131	0.765 (0.535–1.093)	0.141
		rs3213245	AA	182	181	Reference	Reference
			AG	53	66	0.799 (0.527–1.211)	0.289
			GG	3	5	0.597 (0.141–2.534)	0.479
			AG + GG	56	71	0.784 (0.523–1.178)	0.241
*XRCC2*		rs3218544	GG	82	78	Reference	Reference
			AG	114	133	0.815 (0.547–1.214)	0.315
			AA	38	41	0.882 (0.514–1.512)	0.647
			AG + AA	152	174	0.831 (0.569–1.214)	0.338
		rs3218385	AA	172	172	Reference	Reference
			AC	53	73	0.726 (0.481–1.096)	0.127
			CC	9	7	1.286 (0.468–3.530)	0.625
			AC + CC	62	80	0.775 (0.523–1.148)	0.203
		rs3218556	GG	166	149	Reference	Reference
			AG	57	77	0.664 (0.442–0.999)	**0.049**
			AA	11	26	0.380 (0.181–0.795)	**0.008**
			AG + AA	68	103	0.593 (0.406–0.865)	**0.006**
*MSH2*		rs2303424	GG	86	102	Reference	Reference
			AG	117	129	1.076 (0.735–1.574)	0.707
			AA	31	20	1.599 (0.868–2.945)	0.131
			AG + AA	148	149	1.178 (0.817–1.699)	0.380
		rs2303425	TT	147	166	Reference	Reference
			CT	72	62	1.311 (0.874–1.968)	0.190
			CC	15	24	0.706 (0.357–1.396)	0.315
			CT + CC	87	86	1.142 (0.788–1.657)	0.483
*XPD*		rs238419	CC	79	95	Reference	Reference
			CT	101	110	1.104 (0.738–1.651)	0.629
			TT	54	47	1.382 (0.845–2.259)	0.197
			CT + TT	155	157	1.187 (0.818–1.722)	0.366
		rs13181	TT	176	209	Reference	Reference
			GT	56	40	1.663 (1.057–2.614)	**0.027**
			GG	2	3	0.792 (0.131–4.791)	0.799
			GT + GG	58	43	1.602 (1.029–2.493)	**0.036**

The *p* values in bold represent a statistically significant association. All data are adjusted for age, gender, smoking history, cancer histology, family history, and treatment with chemotherapy.

**Table 3:** Association between candidate SNPs and radiation-induced toxic reactions.

Gene	SNPs	Genotype	Low toxic reactions	High toxic reactions	OR (95% CI)	*p*
*XRCC1*	rs25487	CC	188	74	Reference	Reference
		CT	114	69	0.650 (0.435–0.972)	**0.035**
		TT	23	18	0.503 (0.257–0.986)	**0.043**
		CT + TT	137	87	0.620 (0.424–0.907)	**0.013**
	rs25489	CC	257	122	Reference	Reference
		CT	65	38	0.812 (0.515–1.279)	0.369
		TT	3	1	0.424 (0.147–13.83)	0.759
		CT + TT	68	39	0.828 (0.528–1.297)	0.409
	rs1799782	GG	175	91	Reference	Reference
		AG	117	58	1.049 (0.700–1.571)	0.817
		AA	33	12	1.430 (0.705–2.902)	0.320
		AG + AA	150	70	1.114 (0.762–1.630)	0.577
	rs3213245	AA	248	117	Reference	Reference
		AG	74	43	0.812 (0.525–1.255)	0.348
		GG	3	1	1.415 (0.146–13.75)	0.764
		AG + GG	77	44	0.826 (0.537–1.270)	0.383
*XRCC2*	rs3218544	GG	117	49	Reference	Reference
		AG	162	83	0.817 (0.534–1.251)	0.353
		AA	46	28	0.688 (0.387–1.224)	0.202
		AG + AA	208	111	0.785 (0.523–1.177)	0.241
	rs3218385	AA	235	109	Reference	Reference
		AC	78	47	0.770 (0.502–1.180)	0.229
		CC	12	5	1.113 (0.383–3.238)	0.844
		AC + CC	90	52	0.803 (0.533–1.210)	0.293
	rs3218556	GG	238	96	Reference	Reference
		AG	78	52	0.605 (0.396–0.924)	**0.019**
		AA	9	13	0.279 (0.116–0.675)	**0.003**
		AG + AA	87	65	0.540 (0.362–0.805)	**0.002**
*MSH2*	rs2303424	GG	123	52	Reference	Reference
		AG	156	82	0.804 (0.528–1.224)	0.309
		AA	46	27	0.720 (0.405–1.280)	0.263
		AG + AA	202	109	0.783 (0.526–1.168)	0.230
	rs2303425	TT	204	106	Reference	Reference
		CT	104	45	1.201 (0.788–1.830)	0.394
		CC	17	10	0.883 (0.391–1.997)	0.765
		CT + CC	121	55	1.143 (0.769–1.698)	0.508
*XPD*	rs238419	CC	102	43	Reference	Reference
		CT	149	77	0.816 (0.520–1.280)	0.375
		TT	74	41	0.761 (0.451–1.283)	0.304
		CT + TT	223	118	0.797 (0.523–1.213)	0.289
	rs13181	TT	235	130	Reference	Reference
		GT	82	27	1.680 (1.035–2.728)	**0.035**
		GG	8	4	1.106 (0.327–3.745)	0.871
		GT + GG	90	31	1.624 (1.025–2.547)	**0.038**

The *p* values in bold indicate a statistically significant association. All data are adjusted for age, gender, smoking history, cancer histology, family history, and treatment with chemotherapy.

## Data Availability

The statistical data used to support the findings of this study are included within the article.

## Supplementary Materials

Supplementary Materials Supplementary Table 1: genomic function of the ten SNPs in XRCC1, XRCC2, MSH2, and XPD genes.
