# Supplementary material for: Potential Functional Variants in DNA Repair Genes Are Associated with Efficacy and Toxicity of Radiotherapy in Patients with Non-Small-Cell Lung Cancer
Source: J Oncol. 2020 Jun 24;2020:3132786. doi: 10.1155/2020/3132786 (PMC7333048; doi:10.1155/2020/3132786)
Supplement: Supplementary Materials — Supplementary Table 1: genomic function of the ten SNPs in XRCC1, XRCC2, MSH2, and XPD genes. [file 3132786.f1.docx]

Supplement Table 1 Genomic function of the ten SNPs in *XRCC1, XRCC2, MSH2, XPD* genes.

| Gene | SNP | HGVS cDNA | HGVS protein | location | MAF |
| --- | --- | --- | --- | --- | --- |
| *XRCC1* | rs25487 | c.1196A>G | p.Gln399Arg | Exon 10 | 0.74 |
|  | rs25489 | c.839G>A | p.Arg280His | Exon 9 | 0.11 |
|  | rs1799782 | c.580C>T | p.Arg194Trp | Exon 6 | 0.31 |
|  | rs3213245 | c.-77C>T | - | 5' UTR | 0.89 |
| *XRCC2* | rs3218556 | c.*2126C>T | - | 3' UTR | 0.18 |
|  | rs3218544 | c.*499C>T | - | 3' UTR | 0.42 |
|  | rs3218385 | c.-69T>G | - | 5' UTR | 0.12 |
| *MSH2* | rs2303424 | c.2744A>G | p.Gln915Arg | Exon 16 | 0.68 |
|  | rs2303425 | c.-118T>C | - | 5' UTR | 0.17 |
| *XPD* | rs13181 | c.2251A>C | p.Lys751Gln | Exon 23 | 0.10 |
|  | rs238419 | - | - | 1236 bp to transcript | 0.51 |
